# Cervical Cancer Prevention in Rural Areas

**DOI:** 10.5334/aogh.4133

**Published:** 2023-11-01

**Authors:** Indira Zhetpisbayeva, Fatima Kassymbekova, Sholpan Sarmuldayeva, Yuliya Semenova, Natalya Glushkova

**Affiliations:** 1 Department of Public Health and Social Sciences, Kazakhstan Medical University “KSPH”, Almaty, Kazakhstan; 2 Department of Clinical Specialties, Al-Farabi Kazakh National University, Almaty, Republic of Kazakhstan; 3 Nazarbayev University School of Medicine, Nazarbayev University, Nur-Sultan, Kazakhstan; 4 Department of Epidemiology, Biostatistics and Evidence Based Medicine, Al-Farabi Kazakh National University, Almaty, Kazakhstan

**Keywords:** cervical cancer screening, HPV vaccination, rural population, adherence

## Abstract

**Objective:**

Globally, cervical cancer (CC) incidence is higher in rural areas than in urban areas that could be explained by the influence of many factors, including inequity in accessibility of the CC prevention measures. This review aimed to identify and analyze factors associated with a lack of cervical cancer screening and HPV vaccination programs in people living in rural areas and to outline strategies to mitigate these factors.

**Methods:**

The literature search encompassed two focal domains: cervical cancer screening and HPV vaccination among populations residing in rural areas, covering publications between January 1, 2004 to December 31, 2021 in the PubMed, Google Scholar, Scopus, and Cyberleninka databases, available in both English and Russian languages.

**Result:**

A literature review identified 22 sources on cervical cancer screening and HPV vaccination in rural and remote areas. These sources revealed similar obstacles to screening and vaccination in both high and low-income countries, such as low awareness and knowledge about CC, screening, and HPV vaccination among rural residents; limited accessibility due to remoteness and dearth of medical facilities and practitioners, associated with a decrease in recommendations from them, and financial constraints, necessitating out-of-pocket expenses. The reviewed sources analyzed strategies to mitigate the outlined challenges. Possible solutions include the introduction of tailored screening and vaccination campaigns designed for residents of rural and remote locations. New screening and vaccination sites have been proposed to overcome geographic barriers. Integrating HPV testing-based CC screening is suggested to counter the lack of healthcare personnel. HPV vaccination is essential for primary cervical cancer prevention, especially in rural and remote areas, as it requires less medical infrastructure.

**Conclusion:**

Certain measures can be proposed to improve the uptake of CC screening and HPV vaccination programs among rural residents, which are needed to address the higher prevalence of CC in rural areas. Further investigation into cervical cancer prevention in rural and remote contexts is necessary to ascertain the optimal strategies that promote health equity.

## Introduction

Globally, cervical cancer (CC) ranks 5th among the major cancer sites [1]. According to the World Health Organization (WHO) in 2018 the age-standardized incidence of CC ranged from 75.0 per 100,000 women in high-risk countries to less than 10.0 per 100,000 in low-risk countries. The World Health Organization has determined that if the incidence drops to four cases per 100,000 women, CC will no longer be considered a public health problem. With this purpose, in 2020 the WHO presented a strategy, which listed three indicators to be achieved by member countries by 2030: 90% vaccination coverage of girls aged less than 15 years; 70% screening coverage of women aged 35–45 years with high-precision tests; and 90% provision of medical care to women diagnosed with cervical disease (both precancerous alterations and established cancer) [2]. Primary CC prevention covers vaccination of adolescent girls against human papillomavirus (HPV) and screening of women for the presence of dysplastic (precancerous) cites in the uterine cervix.

Population-based screening with the help of HPV testing (Co-test) is perhaps the most effective, but the most resource consuming approach both in terms of financial resources and qualified medical professionals [3]. However, each country selects approach depending on healthcare capacities. It has to be noted that effectiveness of screening programs varies with the level of population engagement (coverage and commitment). It was estimated that in order for a screening to be efficient, at least 70% of the target population has to be covered. This level is not always attained as for a variety of reasons many countries do not cross the 50% threshold [4,5].

Cervical cancer incidence varies not only with geographic area, but also with the place of residence. Such, the disease is 15% less common in urban areas as compared with the rural. Cities also experienced a more notable decline in the cancer incidence (10.2% vs. 4.8% in the rural area). The greatest difference was found in the incidence of cancers associated with modifiable risk factors, like tobacco smoking, HPV infection, and availability of screening programs [6]. Besides, rural populations often face disparities in terms of cancer prevention strategies, which is manifested by lower coverage with both CC screening and HPV vaccination [7]. The reasons for this disparity are complex and may include:

1. geographical and socio-economic barriers in obtaining medical care
2. lack of recommendations from the side of medical workers
3. low awareness of cervical cancer and HPV infection
4. low awareness of and commitment to screening for CC and HPV vaccination
5. socio-cultural barriers against application for gynecology services and vaccination of girls
6. limited access to diagnostic and curative services for pre-malignant conditions, etc [8].

Commitment of women residing in rural areas to get CC screening is most weak in rural areas, since women are often unaware about the potential threats of CC. It has to be noted that people residing in rural areas may be socioeconomically deprived and have inadequate hygiene standards and poor sanitation. Also, women living in rural areas may be exposed to other risk factors, like early marriages and multiple pregnancies, which make them more susceptible to CC. Moreover, many rural areas around the globe face a lack of medical and social facilities and this limits the possibility of obtaining sound advice and guidance. Under such conditions, various strategies to improve screening, like establishment of rural cancer registries, have proven useful in minimizing the magnitude of this public health problem [6]. The implementation of self-sampling for HPV DNA testing, as opposed to traditional cytological screening, has the potential to significantly impact the challenge of improving cervical cancer screening coverage in rural areas [9]. Thus, this review is aimed at comprehensive analysis of the range of issues related to the primary and secondary prevention of CC in rural areas of the world, including CC screening and HPV vaccination.

## Materials and Methods of Research

### Search strategy

To meet the review aim, a thorough search of literature was carried out in the following databases: Scopus, PubMed (Medline), Google Scholar, and Cyberleninka. The search strategy aimed to identify relevant studies regarding cervical cancer screening and human papillomavirus (HPV) vaccination in rural areas. Search parameters were limited to studies published between January 1, 2004 and December 31, 2021 The search strategy utilized a combination of Medical Subject Headings (MeSH) terms, including [“Uterine Cervical Cancer” (MeSH)] and [“Cancer Screening Test” (MeSH)] or [“Human Papillomavirus Vaccine (MeSH)], and [“Rural Population” (MeSH)]. No restrictions were imposed regarding the selection of countries or their income levels. The list of selected studies was composed and checked for the presence of duplicates, which were eliminated.

### Study selection and screening

The initial screening process began with a review of the titles of retrieved papers to determine their relevance to the scope of this review. The search included studies where the study participants were people living in rural or remote areas, and the design of these studies was descriptive, including qualitative and quantitative methods, observational, and interventional with the evaluation of educational interventions. Exclusion criteria encompassed unavailability of full text or full text in languages other than English or Russian, content falling outside the scope of the review’s aim, publications outside the specified time frame, and studies with poor methodological quality, such as commentaries, editorials, case reports, and correspondence letters. Subsequently, abstracts were retrieved and evaluated to confirm if a study met the inclusion criteria (Table 1). Next, the papers’ abstracts were obtained and it was ascertained that they: (i) reported the utilization of CC screening conducted among women aged 9 and 70 years old; (ii) evaluated the HPV vaccination related issues; (iii) focused on population residing in rural areas; and (iv) published in English or Russian languages.

**Table 1:** Inclusion and exclusion criteria of study selection.


INCLUSION CRITERIA	EXCLUSION CRITERIA

Original papers describing all methods of CC screening in rural areas among women aged 20 to 70 years old	Studies falling outside the scope of the review’s aim.

Original papers describing HPV vaccination in rural areas	Studies examining CC screening and HPV vaccination without the place of residence specification

Articles published between January 1, 2004 to December 31, 2021	Studies on HPV vaccination among boys

Full text articles	Studies examining HPV infection

	Studies investigating vaccines other than HPV

	Unavailability of full text studies

	Duplicate of papers


**Figure 1:**
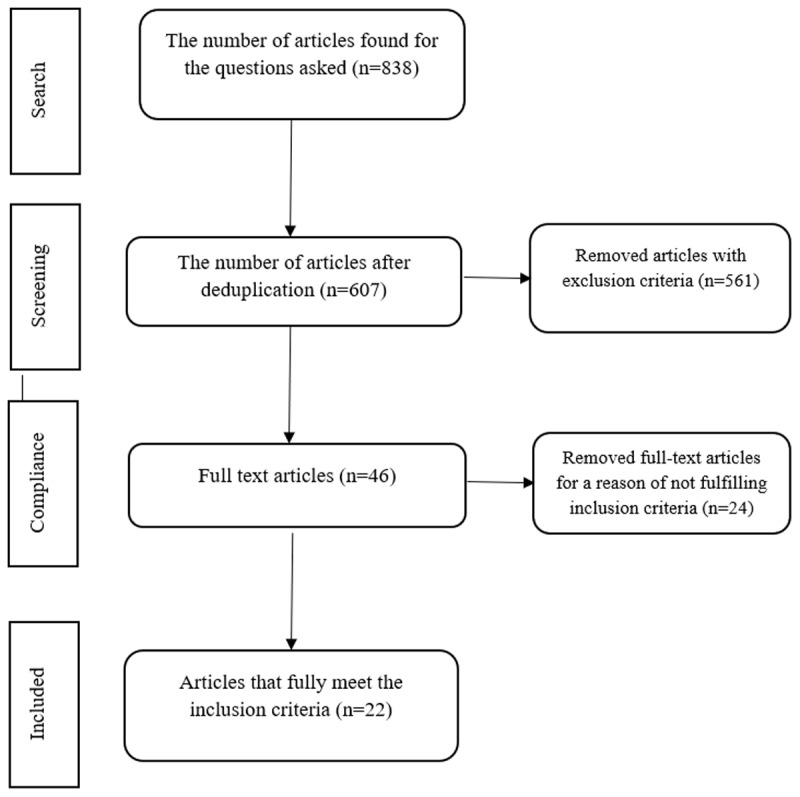
Article selection flowchart.

Studies failing to fulfill the inclusion criteria were excluded. Article selection flowchart is presented in Figure 1.

The initial search from the databases included 838 articles. Following duplicate removal 607 were eligible for the screening process, and a total of 22 articles fulfilled the study criteria and were included in this review. The resulting manuscript was structured in a form of narrative review and was discussed between all co-authors.

Given the study’s design and objectives, it wasn’t feasible to conduct a comparative analysis of cervical cancer prevention challenges in urban and rural areas, which could be a potential limitation, as densely populated urban regions in some countries might share similar constraints in accessing screening services with women in rural areas. This review analyzed the prevalent barriers encountered by cervical cancer prevention initiatives in rural and remote regions across diverse countries with varying capacities. A potential limitation of this study is that the proposed solutions may not universally apply to diverse settings.

#### International experience on implementation of CC screening programs in rural areas

Nowadays, CC screening programs are actively implemented and widely used by different healthcare systems across the globe. Nevertheless, these screening programs are not always successful as they are dependent on such factors as population adherence and coverage. Meanwhile, it is important to measure the population adherence since it can help to identify “weaknesses,” the spots of non-effectiveness, to overcome them. Besides, interventions targeted on elimination of inequalities must be envisaged and for this, inequalities need to be defined and traced. Addressing the issue of inequality in CC screening availability concerning residential location, service accessibility, and economic standing of regions, it is essential to consider that a primary approach to enhance CC screening program coverage within low-resource settings involves transitioning from routine cytological CC screening (Pap test) to self-sampling and HPV DNA testing, which is a more cost-effective method. This transition aligns with the recommendations by the WHO for CC screening [10].

There is a range of international studies investigating the problems associated with implementation of CC screening programs in rural areas and proposing possible solutions (Table 2).

**Table 2:** International experience on implementation of CC screening programs in rural areas: major problems and possible solutions.


AUTHORS(YEAR OF PUBLICATION)	COUNTRY	STUDY POPULATION	TYPE OF CC SCREENING	STUDY DESIGN	KEY FINDINGS	PROPOSED SOLUTIONS

Liu et al (2017) [9]	China	Women aged 35–64 years	Population-based cervical cancer screening (National Cervical Cancer Screening Program in Rural Areas)	Survey	The vast majority of women (96,0%) expressed positive attitudes towards screening. Still, many respondents reported low awareness of the screening program, and more than a third (36,3%) had never taken part in the program	Information campaigns among target population group. Teaching medical personnel about C identification via screening. Mechanisms to ensure the continuity of health education should be envisaged.

Thompson et al (2017) [14]	Latin America	3 years after the age of initiation of sexual activity	Population-based cervical cancer screening	Randomized controlled trial with educational interventions	Women living in rural areas, low socioeconomic status and high enclave areas have 12.7 times higher rates of invasive CC than those who live in areas of high socioeconomic status and low enclave areas. More than 60% of late-stage cancers are found in the areas with low health care and under-examined groups of women.	women residing in rural areas

Ndejjo et al (2016) [24]	Uganda	Women aged 25–49 (VIA, 3 years); 30–49 (HPV)	National Cervical Cancer Screening recommendations	Survey	Of the 900 women, only 43 (4.8%) had ever been screened for CC. Barriers to cervical cancer screening were negative individual perceptions 553 (64.5%) and health facility related challenges 142 (16.6%).	Increase access to cervical cancer screening in rural areas and engage health workers to discuss the CC disease with women.

Ruddies et al (2020) [22]	Ethiopia	Women aged 30–49 years	No organized or opportunistic cervical cancer screening program	Survey	Only eight women (2.3%) had been screened before. Although 240 women (70.4%) had the intention to be screened, only 107 (31.4%) said that they had access to a screening facility. Living in an urban setting made it 3.35 times more likely to have a positive attitude towards cervical cancer screening as compared with women living in rural areas.	Special emphasis should be put on training of health care providers with a focus on cervical cancer and its screening,

Rosser et al (2015) [29]	Kenya	Women aged 25–49 (VIA, 5 years); 25–30 (cytology, 5 years); 30–49 (HPV test, 5 years)	National Cervical Cancer Screening Program. Pilot implementation of self-sampling HPV testing	Survey	The main obstacles in providing services were a lack of sufficient staff (62%), inadequate training or a shortage of trained personnel (60%), low staff motivation (25%), insufficient space for screening activities (35%), and difficulty with supplies (31%) or autoclaving (9%). Also, low community mobilization as a problem within the population	Additional health care providers training, increased community mobilization by educational campaigns and training for both groups

Gottschlich et al (2021) [30]	Guatemala	Women aged 25–29 (cytology, 3 years); 50–54 (cytology, 3 years); 30–49 (cytology, 3 years); 30–39 (HPV test, 5 years); 40–49 (VIA, 3 years)	National Cervical Cancer Screening Program.	Qualitative, in-depth interview	Barriers to screening included ancillary costs, control by male partners, poor provider communication and systems-level resource constraints, like shortages of tests and long wait times	Discussions with women who have been screened for cervical cancer, health campaigns, self-screening for HPV


East China is one of the places with a heavy burden associated with CC. Although the Chinese government continuously provides affordable, free CC screening to women residing in rural areas aged 35–64 years, the program has low coverage even in more developed parts of East China. The authors posit that a lack of awareness regarding CC screening among women residing in rural areas constitutes the primary issue leading to inadequate coverage. Furthermore, they underscore the pivotal role of healthcare workers in mitigating this challenge. The main problem affecting low attendance in screening is low awareness of the existing screening program. According to the results of this study, over a third of women living in rural areas have never participated in cervical cancer screening. However, the overwhelming majority of women in rural areas have a positive attitude towards screening. Another important factor is the role of healthcare workers, as they contribute to health promotion and provide information about CC and CC screening. Thus, the knowledge of medical professionals about CC is very important since they have to provide accurate and up-to-date information to women. Therefore, it was proposed to target the primary healthcare professionals with upgraded training on a range of issues related to CC. In addition, different approaches to ensure the continuity of health education should be studied and implemented, as one short speech on health issues may not transform into improved screening attendance. Despite the fact that education-oriented approach is relatively costly and time consuming, it is likely to have a long-lasting impact, which will manifest as reduced mortality and improved survival of CC patients [11].

Certain Latin American countries demonstrate the highest incidence and mortality rates from CC (9.4 and 2.6 per 100,000 people, respectively). These numbers even surpass the data observed for Afro-American population (8.9 and 3.9 per 100,000 people, respectively) [12,13]. Perhaps, one of the contributing factors to this alarming situation is a relatively low level of CC screening in rural areas of Latin America. Such, those Latin American women who live in rural areas, have 12.7 times higher rates of invasive CC than those who are living in areas of high socioeconomic status [14]. Also, more than 60% of late-stage cancers are found in the places with low level of healthcare provision, which is common for rural regions [15]. Consequently, Latin American women of lower educational and socioeconomic status living in rural areas and enclaves are significantly less likely to be screened for CC than other Latin American women. Bearing in mind that the proportion of rural population in Latin America is high, there is a need to increase adherence to CC screening appointments among rural communities, which could be done via introduction of educational interventions that are grounded on the “promoter” program [12].

There is definitely a need for specific, clear policy measures targeted at raising the CC screening coverage among rural populations. For this, certain interventions could be proposed which address each of the sensitive issues: reaching those who are underserved, increasing awareness of target population groups and sensitizing policy makers on these issues. As a result, several different strategies have been suggested to improve the screening behavior. These strategies include preparation and sending of reminders, provision of various educational campaigns [16,17], elimination or reduction of structural and financial barriers [18], and activities aimed at improving knowledge of CC screening among the medical professionals. Besides, it is worth developing recommendations on the use of individualized educational interventions, to encourage and motivate women to undergo the CC screening [19,20] and specifically adapt all interventions to the needs of specific population groups.

The HPV self-sampling campaign implemented in Bolivian rural regions effectively elevated screening coverage, achieving the annual average within a mere three-month period [21].

Therefore considering the constraints of limited resources, it is advisable to explore alternatives to routine cytological screening, as suggested in the recent WHO recommendations: implementation of HPV DNA testing and self-sampling as the preferred methods in remote and rural areas [9].

Peru exhibits a high incidence of cervical cancer, also, there is a low level of CC screening coverage. In Peru inadequate screening is due to low public awareness of cervical cancer and the HPV vaccine. This study emphasizes medical professionals’ views, highlighting the negative perception of healthcare services and the absence of a culture of preventive examinations by population. Addressing the issue of limited coverage necessitates educational initiatives in rural Andean Peru. These campaigns are indispensable for increasing awareness about cervical cancer (CC) and its screening, employing materials that align with the cultural context [22].

Additionally, another study conducted within a rural population in Mexico emphasized organizational obstacles to cytology screening, including irregular material supply, distant clinic location, and inadequate communication between staff and patients. Women were provided with the option of self-sampling for HPV. Participants perceived this approach as simpler, less embarrassing, and less painful than cytology. Shifting to HPV self-testing rather than cytology may mitigate certain gender, organizational, or technical quality of care concerns [23].

Several studies conducted in rural areas of African countries such as Uganda, Malawi, Ethiopia and Kenya have also identified major barriers to CC screening. Cervical cancer poses a significant threat to women’s health in Uganda. In 2010, Uganda launched a strategic plan to prevent and manage cervical cancer. However, in rural areas, CC screening coverage remains low due to limited awareness, healthcare challenges, individual perceptions, lack of visible symptoms, low risk perception, time constraints, and test result apprehensions. To address these challenges, improving access to cervical cancer screening in rural areas and engaging healthcare professionals in proactive discussions with women, emphasizing screening awareness, thus increasing their adherence to CC screening, is crucial [24].

A study conducted in Malawi found that the main barriers to CC screening were low knowledge, perceived low susceptibility. Study participants did not perceive CC screening as essential healthcare and typically underwent screening when seeking medical assistance for gynecological issues. It is essential for healthcare providers to prioritize improving patients’ understanding of cervical cancer and their capacity to evaluate their individual risks. Moreover, consistent support and active promotion of cervical cancer screening are of paramount importance. These measures could present an optimal solution to the issue of cervical cancer (CC) screening in rural Malawi [25].

Ethiopia is one of the developing countries where cervical cancer has high incidence and mortality rates, and access to screening and treatment, knowledge about HPV and cervical cancer is limited. These barriers contribute to women’s low susceptibility to cervical cancer, which in turn is reflected in inadequate screening practices. Among Ethiopian women in rural areas, a positive attitude towards screening is formed by the influence of socio-demographic factors. Often, women with a higher level of education, who are aware of cervical cancer and use contraceptives, have a higher adherence to screening. It should be noted that educational interventions are needed in rural Ethiopia regarding adequate information on risk factors, screening and its availability. Considering that medical personnel are the main source of information about cervical cancer and its screening, it is very important to conduct their continuous training in these matters [26]. Another study conducted in Ethiopia emphasized the introduction of HPV self-sampling as a significant solution to address the challenges of accessibility and low coverage in cervical cancer (CC) screening. To enhance its effectiveness, the authors emphasize the importance of raising awareness, mobilizing the community, and involving families in this process [27]. In Kenya, the main problems of low coverage include inadequate staffing, a shortage of adequately trained personnel or insufficient training, limited staff enthusiasm, inadequate facilities for screening and difficulties in obtaining supplies or performing autoclaving [28]. The solutions to these problems include additional health care providers training, increased community mobilization by educational campaigns and training for both groups [29]. In Guatemala, the scarce availability of efficient screening and treatment options has led to significantly elevated rates of cervical cancer incidence and mortality. A study conducted in Guatemala, assessing the integration of HPV self-sampling, indicates that introducing this program in low-income populations, particularly within predominantly indigenous and rural communities, could enhance engagement with established cervical cancer screening programs [30].

#### Vaccination against human papillomavirus in rural areas

Although CC screening program requires significant infrastructural and organizational investments, HPV vaccination sets fewer logistical demands on the healthcare system than repeated screening, testing, and treatment for cervical disease. This approach is considered to be extremely important in the light of the primary prevention of CC in rural areas.

However, provision of HPV vaccination to the rural population is associated with certain difficulties that result low coverage. Such, when comparing coverage with HPV vaccination in the United States it was found out that the chances of starting vaccination were lower in the villagers by almost 15% as compared with the urban dwellers [31]. In rural areas it is important to set diverse strategies to overcome geographical, communicational, and other barriers at various levels: patient, medical organizations, community, state, and country. Such measures include changing and adapting organizational processes, evaluating the performance of individual clinics and healthcare workers, provision of educational programs, setting up vaccination in schools, pharmacies, and public places. Besides, for a HPV vaccination program to be effective, local characteristics have to be taken into account to adapt communication strategies and this necessitates research on what works especially well in rural areas [8].

Numerous studies have confirmed the relationship between the level of HPV awareness, its association with CC, knowledge about availability of effective vaccine, and the intention to be vaccinated among various populations. It was not surprising that better awareness was associated with higher levels of education and older age [32,33,34,35]. The place of residence also plays role and such, for example, in the Mysore region of India urban parents were more than twice as knowledgeable about HPV, CC, and HPV vaccinations as rural parents [36]. Another study from China has shown that urban residents had heard about HPV much more often than the rural residents (39.1% vs. 27.1%, respectively). Also, they were better informed about the HPV vaccine (23.7% vs. 15.1%, respectively). Moreover, women with a higher knowledge more often expressed a positive opinion about vaccination [37].

An interview-based study from Malaysia found an extremely low knowledge of women residing in rural areas aged 18–25 years about HPV, cervical cancer, and the vaccine. This knowledge was so low that an average score equaled 2.4 points out of 14. The intention to be vaccinated was associated with awareness of screening and CC risk factors [38]. Similar data were obtained in a study coming out from a rural area in China’s Hong Nan province, where 58.8% of women aged 20–45 years showed the intention to be vaccinated. Older age and higher educational level were associated with the intention to be vaccinated and women who were aware of the HPV vaccine and that CC is a preventable disease, expressed the desire for vaccination two times more often than those who were not informed. Meanwhile, women who had never heard of the vaccine and were worried about the possible side effects were more likely to refuse vaccination [39]. Several studies from the USA also confirmed the fact that rural residents are less informed about HPV and HPV vaccination [40,41].

The studies conducted in the Commonwealth of Independent States show that local parents are often vaccine hesitant, and this impacts vaccination uptake rates which are especially low in rural areas. For instance, in Russian Federation rural parents are more likely to refuse vaccines as compared with the urban parents (17% vs. 12%) [42]. In the Republic of Kazakhstan, there is low awareness of parents about availability of HPV vaccines (66% ever heard about this) and medical workers and the Internet serve as the main information sources. Like in case with China, a positive decision to vaccinate against the HPV was associated with older age and higher level of education. Nevertheless, there were no significant differences in awareness of HPV and the HPV vaccines among rural and urban residents [43]. Another study from Kazakhstan also failed to reveal the relation between the place of residence and parental vaccine hesitancy [44]. Table 3 summarizes the major finding of international studies on the knowledge of HPV vaccination in different population groups.

**Table 3:** Knowledge of HPV and HPV vaccination in rural different population groups across the globe.


AUTHORS (YEAR OF PUBLICATION)	COUNTRY	STUDY POPULATION	STUDE DESIGN	AWARENESS ABOUT HPV VACCINATION SS

Ping Wong et al (2010) [38]	Malaysia	Young women residing in rural areas in Malaysia were interviewed using a standard questionnaire (N = 449).	Survey	The mean total knowledge score (14-item questionnaire) was 2.37 (SD±1.97). Although many respondents never heard of the HPV vaccine, two-thirds professed an intention to receive the HPV vaccine. Intention to receive the vaccine was significantly associated with knowledge of cervical screening and cervical cancer risk factors.

Thomas et al (2012) [58]	USA	African American parents or caregivers with children 9–13 years of age completed a survey (N = 400).	Survey	Perceived vulnerability (knowledge about HPV) constituted 40.4%, while perceived severity (awareness that HPV can cause a CC) equaled 45.6%.

Feng et al (2012) [37]	China	Women attending the checkup clinics were invited to complete a questionnaire-guided interview (N = 1432).	Qualitative, interview	39.1% of women living in urban areas and 27.1% of women in rural areas were aware about HPV, whereas 23.7% and 15.1%, respectively, heard of the HPV vaccine. The mean score of HPV knowledge was 3.75 in residents of urban areas and 3.18 in residents of rural areas.

Blake et al (2015) [59]	USA	National Cancer Institute’s 2013 Health Information National Trends Survey of USA adult, civilian, non-institutionalized people (N = 3185).	Survey	People living in rural areas were significantly less likely to know that HPV causes cervical cancer as compared with those living in urban areas.

Nasritdinova et al (2016) [43]	Kazakhstan	Population of four regions of Kazakhstan took part in anonymous survey (N = 5338)	Survey	66% of respondents were aware about existence of HPV vaccine. No significant difference between urban and women residing in rural areas was detected.

Boyd e al (2018) [41]	USA	Vaccinated and non-vaccinated adolescents aged 11–18 years and their caregivers from three rural counties of south Alabama participated in individual interviews (N = 48).	Qualitative, interview	75% of caregivers and 33% of adolescents heard about HPV and 62.5% of adolescents were aware that HPV can lead to cervical cancer as compared with 55.6% of the caregivers. 60% of caregivers of non-vaccinated adolescents and 33.3% caregivers of non-vaccinated adolescents heard about the HPV vaccine.

Mohammed et al (2018) [40]	USA	Respondents older than ≥18 years completed the Health Information National Trends Survey 2013–2017 (N = 10147).	Survey	55.8% and 58.6% of rural residents were aware of HPV and HPV vaccine, respectively. As compared with urban residents, rural residents were less likely to be aware of HPV and HPV vaccine. Rural residents were less likely to know that HPV causes cervical cancer, and that HPV can be transmitted through sexual contact.

Degarege et al (2018) [36]	India	Parents of school-going adolescent girls completed a self-administered questionnaire (N = 1609).	Survey	Urban parents were more likely to believe that both HPV infection and CC could cause serious health problems. Parents’ belief that HPV vaccination will make girls sexually active was lower among urban parents as compared with rural. There was no significant difference between urban and rural parents in beliefs about susceptibility of their daughters to HPV infection or cervical cancer, and beliefs about the safety and ability of HPV vaccine to protect against cervical cancer.

Touch and Oh (2018) [54]	Cambodia	Women aged 20–69 years who lived in Kampong Speu Province participated in the survey (N = 440).	Survey	Only 2% of women were aware that HPV infection is a risk factor for cervical cancer; 8.6% of women were aware that HPV is a sexually transmitted infection; 35.2% of women knew that cervical cancer can be prevented by vaccination; and 62% of women were willing to receive vaccination for themselves as well as for their daughters.

Qin et al (2020) [39]	China	Women aged 20–45 years from rural areas of Hunan Province in China completed the anonymous self-administered questionnaire (N = 2101).	Survey	21.6% of women were aware of HPV as a risk factor of CC and 50.28% of women knew about HPV vaccine.

Banik et al (2020) [46]	Bangladesh	Women of reproductive age living in rural areas of Bangladesh were interviewed with a semi-structured questionnaire (N = 600).	Survey	55.2% of respondents identified HPV infection as a risk factor for CC, and 48.3% knew that HPV vaccine can prevent CC.

Kadian et al (2020) [60]	India	Women of urban and rural background aged 18–65 years completed the questionnaire (N = 1500)	Survey	55% of women had little knowledge about cervical cancer, and 87.5% were informed about HPV infection, while 95% were aware about HPV vaccine. Good knowledge about HPV infection and HPV vaccination was very low in both rural (6.25% and 1.25%, respectively) and urban (14.3% and 4.3%, respectively) areas.


#### Sources of information used by rural people to get knowledge about cervical cancer, HPV, and HPV vaccination

Since many studies reported low levels of awareness about strategies used to prevent CC, it is necessary to focus on the sources of information used by different people in order to increase their vaccine literacy. In rural areas of Cambodia, the media, i.e., radio and television, was recognized to be the most common information source (39%). The reason behind this is the availability of radio and TV sets at homes, which underlines the undoubted importance of disseminating health information through these sources. Much less often, the villagers received information from medical workers or medical organizations (10%) [45]. A study performed in the rural areas of Bangladesh also demonstrated that the media is the most popular information source (53.4%), followed by medical professionals (35.3%), the Internet and social networks (30.4%), family members (23.7%), friends and neighbors (14.5%) [46]. Female residents of villages in China named medical workers as the most trusted source of information (58.8%), and thereafter were called WeChat, microblogs, TV programs, and the Internet [39].

Lack of advice from the side of health workers is one of the main reasons for the decline in vaccination coverage in rural areas of the United States and this includes inappropriate notification [47,48]. The advice of a qualified health professional plays a significant role for parents when making decision on vaccination of their children. It has been proven that a strong recommendation from a doctor can increase the level of vaccination uptake by three to nine times [49]. It has to be recognized that rural healthcare experiences a shortage of medical staff and when this is coupled with a substantial heterogeneity of patients, it leads to the insufficient knowledge about adolescent immunization. Still, rural population tends to trust the doctor’s opinion more than urban [50].

In the Russian Federation, a great proportion of rural parents trust their local doctors (91.7%), but 71.2% of them expressed the need for additional information. Similar findings were obtained in the Kyrgyz Republic, where 72.8% of rural mothers trust the opinion of doctors, but they also were willing to receive additional information [51]. In the Republic of Kazakhstan the level of trust in medical workers expressed by parents when making decision about mandatory childhood vaccinations was 68.1% among those who agreed to vaccinate, while those who refused to do so, trusted the Internet more [44]. Table 4 in Supplementary Materials presents the main findings on the sources of information about HPV vaccination used by members of different communities across the globe.

#### Availability of infrastructure for the HPV vaccination in rural areas and considerations about the cost

Lower coverage rates with the HPV vaccination in rural areas can also be attributed to the lack of access to transportation, which occurs in both developed and developing countries. Such, in the United States rural parents often delay vaccination because of transport inaccessibility [45]. Likewise, developing countries of Africa face the problem with transport accessibility as one of the existing barriers for vaccination, which is significantly more pronounced in rural areas than in the cities (27% vs. 12%) [52].

Depending on the possibilities available within the country, different countries solve this problem in different ways. The problem of transportation to healthcare facilities for the HPV vaccination can be overcome in the following ways: provision of vaccination in schools, pharmacies, dental clinics, arrangement of mobile vaccination clinics, involvement of social workers, and development of navigation schemes for parents.

Although setting a vaccination program in a medical facility has clear advantages that are related to the provision of quick assistance when needed as well as advice from qualified medical personnel, this is not always possible in rural areas. Thus, in the United States it was proposed to provide vaccination in rural pharmacies. The rationale behind this decision is that pharmacists often enjoy the same level of trust from local residents as other medical professionals do and are the most accessible. This is, in particular, due to their proximity, a wide network of pharmacies across the country, convenient opening hours, and absence of the need to make an appointment in contrast with the clinics. A vaccination program on the basis of an existing pharmacy network can help to overcome the structural barrier at the patient level, which also includes lack of time, financial restraints, and unavailability of transportation. Of interest is the fact that an interview-based study on caregivers of adolescents in rural areas of the United States demonstrated a low awareness about the possibility to get vaccination in local pharmacies. Still, most respondents considered a pharmacy to be a more convenient place for vaccinations, which saves their time and money [53].

Another structural barrier for the HPV vaccination in rural areas is the cost. The HPV vaccine is still one of the most expensive vaccines available. Despite significant reductions in vaccine prices for low- and middle-income countries, the cost remains prohibitively high with considering additional expenditures imposed on residents of rural and remote areas. For instance, the rural population of Cambodia showed high motivation for the HPV vaccination, but lack of knowledge and the vaccine cost have become the major barriers for uptake of the HPV vaccine [54]. The study carried-out in rural Bangladesh also found a high level of intention to get vaccinated, but vaccination coverage remains extremely low (5.3%). Like in case with Cambodia, the main reasons for this phenomenon are the high cost of vaccine (40.1%) and the lack of knowledge (34.3%) [46].

The full economic cost of the vaccination program includes the cost of the HPV vaccine, but also other costs associated with the program planning, staff training and mobilization, delivery of the vaccine, organization of storage, and provision of cold chain. These costs make up about 47% of the total economic cost [55]. In this regard, an important role in achieving optimal coverage is played by the financial availability of vaccination, in particular, full coverage at the expense of the state or insurance companies. However, in several countries vaccination against HPV is carried out on a paid basis, which is certainly an obstacle to obtaining a desirable level of vaccination among the population. The study from Vietnam showed that rural residents were almost 10 times more interested in vaccination than city dwellers. However, after the vaccination price was articulated, the desire to get vaccinated decreased dramatically [56]. The study from rural China found out that 8.5% of women cited high costs as a barrier to vaccination [39].

**Figure 2:**
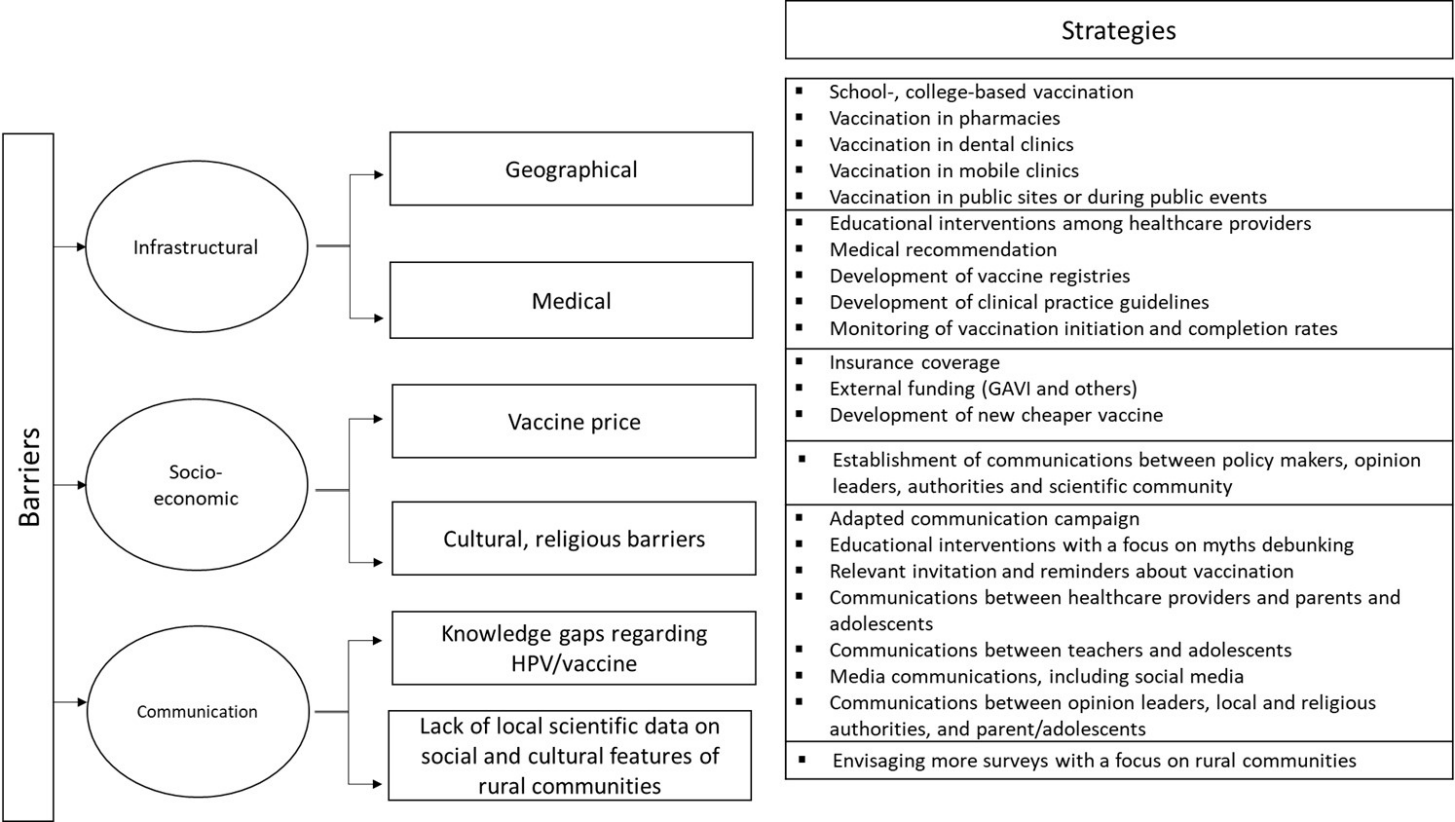
Strategies to overcome the infrastructure, communication, and cost-related barriers in rural areas.

Meanwhile, financial support for low- and middle-income countries could be provided by the Global Alliance for Vaccines and Immunizations (GAVI), sponsored by some governments and private foundations. By 2019, 19 countries (35% of all middle- and low-income countries) received financial support from the GAVI. Funding comes from a grant whereby the cost of the vaccine for a cohort of nine-year-old girls could be as low as 2.40 United States dollars in the first year of vaccine introduction and the grant also covers necessary staff training. In addition, during the first year, the Alliance covers the costs of vaccination of a cohort of girls aged 10–14 years [57]. Figure 2 presents a summary of strategies that could be implemented to overcome infrastructure and cost-related barriers in rural areas.

## Concluding Remarks

There are substantial inequalities in access to and uptake of CC screening and HPV vaccination between urban and rural populations. This may be explained by unavailability or inaccessibility of medical services, lower socio-economic status, and medical ignorance, which exist in many countries and are particularly common among the rural population. Nevertheless, certain interventions could be proposed to improve the CC prevention programs in the rural areas and these include conducting widely implementation of HPV DNA testing (including self-sampling testing), educational interventions among the target groups of women and healthcare professionals involved in CC screening and HPV vaccination programs. Besides, there is a need to increase availability of the HPV vaccination by means of subsidizing the vaccine cost, but also raising awareness of the rural population and improving accessibility through the provision of shots in proximity to the place of residence.

## Additional File

The additional file for this article can be found as follows

10.5334/aogh.4133.s1 Supplementary Materials. **Table 4.** Sources of information about CC, HPV and HPV vaccination as reported by members of different communities.
