# Supplementary material for: Cervical Cancer Prevention in Rural Areas
Source: Ann Glob Health. 2023 Nov 1;89(1):75. doi: 10.5334/aogh.4133 (PMC10624144; doi:10.5334/aogh.4133)
Supplement: Supplementary Materials. — Table 4. Sources of information about CC, HPV and HPV vaccination as reported by members of different communities. [file agh-89-1-4133-s1.pdf]

Supplementary materials.

Table 4. Sources of information about CC, HPV and HPV vaccination as reported by members of different communities

Authors (year of publication)	Country	Study population	Sources of information
Boyd e al (2018) [42]	USA	Vaccinated and non-vaccinated adolescents aged 11–18 years and their caregivers from three rural counties of south Alabama participated in individual interviews (N=48).	Healthcare provider was the most popular sources of information about HPV, followed by TV, teachers and parents.
Kadian et al (2020) [61]	India	Urban and women residing in rural areas aged 18-65 years completed the questionnaire (N=1500)	School/college staff (70 %), friends/neighborhood/relatives (50,1 %), medical practitioners (44,5 %), newspapers (38,2 %), television (30,7 %) and Internet (19 %) were the main sources of information in rural area.
Touch and Oh (2018) [55]	Cambodia	Women aged 20–69 years who lived in Kampong Speu Province participate in the survey (N=440).	Family member / relative/ friend/ school staff/ NGO/ missionary/health magazine were named by 50 % of women as information sources, while 39,4 % named radio and TV, and only 9,8 % named medical/ hospital staff.
Banik et al (2020) [47]	Bangladesh	Women of reproductive age living in rural areas of Bangladesh were interviewed with a semi-structured questionnaire (N=600).	53.4% of women heard about cervical cancer from mass media (i.e., Television, Newspaper, Radio), followed by health care providers (35.3 %), internet and social media (30.4 %), family members (23.7 %), neighbors/friends (14.5 %), and from books or other written sources (2.6%).
